# Marsh macrophyte responses to inundation anticipate impacts of sea-level rise and indicate ongoing drowning of North Carolina marshes

**DOI:** 10.1007/s00227-012-2076-5

**Published:** 2012-10-17

**Authors:** Christine M. Voss, Robert R. Christian, James T. Morris

**Affiliations:** 1 Coastal Resources Management, East Carolina University, Greenville, NC 27858 USA; 2 Present Address: Institute of Marine Sciences, University of North Carolina at Chapel Hill, Morehead City, NC 28557 USA; 3 Department of Biology, East Carolina University, Greenville, NC 27858 USA; 4 Department of Biological Sciences and the Belle W. Baruch Institute for Marine and Coastal Sciences, University of South Carolina, Columbia, SC 29208 USA

## Abstract

In situ persistence of coastal marsh habitat as sea level rises depends on whether macrophytes induce compensatory accretion of the marsh surface. Experimental planters in two North Carolina marshes served to expose two dominant macrophyte species to six different elevations spanning 0.75 m (inundation durations 0.4–99 %). *Spartina alterniflora* and *Juncus roemerianus* exhibited similar responses—with production in planters suggesting initial increases and then demonstrating subsequent steep declines with increasing inundation, conforming to a segment of the ecophysiological parabola. Projecting inundation levels experienced by macrophytes in the planters onto adjacent marsh platforms revealed that neither species occupied elevations associated with increasing production. Declining macrophyte production with rising seas reduces both bioaccumulation of roots below-ground and baffle-induced sedimentation above-ground. By occupying only descending portions of the parabola, macrophytes in central North Carolina marshes are responding to rising water levels by progressive declines in production, ultimately leading to marsh drowning.

**Electronic supplementary material:**

The online version of this article (doi:10.1007/s00227-012-2076-5) contains supplementary material, which is available to authorized users.

## Introduction

Coastal marshes have been identified as habitats at high risk of loss and functional degradation (Scavia et al. 2002; Peterson et al. 2008) from accelerating sea-level rise (IPCC 2007; Kemp et al. 2011). Yet coastal wetlands have a record of maintaining elevation relative to sea level for millennia (Redfield 1965, 1972) through both vertical accretion of the marsh surface and horizontal expansion across the landscape through transgression and progradation (Redfield 1972; Orson et al. 1987; Reed 2002). Marsh macrophytes mediate accretion processes: emergent above-ground vegetation helps trap sediment particles by baffling water flow and thereby enhancing sedimentation (Leonard et al. 1995; Mudd et al. 2010), while below-ground roots and rhizomes add organic matter directly to the soils and result in marsh surface accretion (Turner et al. 2000; Blum and Christian 2004; Nyman et al. 2006; Mudd et al. 2009). How effectively macrophytes facilitate vertical accretion of the marsh surface depends on the plants’ ecophysiological response to inundation (Turner et al. 2000). Thus, interacting relationships among marsh surface elevation relative to sea level, marsh hydrology, and macrophyte responses determine the ecophysiological performance of marsh macrophytes and in situ persistence of the marsh ecosystem (Morris et al. 2002; Kirwan et al. 2010).

*Spartina alterniflora* (hereafter *Spartina*) and *Juncus roemerianus* (hereafter *Juncus*) comprise the dominant macrophytes (Eleuterius 1976; Mitsch and Gosselink 2000) in 90% of U.S. coastal marsh habitat, the majority of which is found along the Atlantic and Gulf of Mexico coasts (NOAA 1990; Watzin and Gosselink 1992). *Spartina* occurs in the upper half of the tidal frame (McKee and Patrick 1988), characteristically forming dense monocultures. Above-ground production of *Spartina* decreases at higher elevations where flooding is less regular (Bertness and Pennings 2000) and increases with increasing inundation until an optimum depth (depth below mean high water) is exceeded (Morris et al. 2002; Morris 2007). With astronomically regular flooding, *Juncus* tends to dominate at slightly higher elevations; while under irregularly flooded conditions of meteorologically dominated forcing, it can dominate the entire marsh (Woerner and Hackney 1997; Brinson and Christian 1999). In field and greenhouse experiments in Georgia, Pennings et al. (2005) showed that *Juncus* is limited by physical stresses (flooding and salinity) at its lower margin and not by interspecific competition, whereas *Spartina* is limited at its landward boundary by competition with *Juncus*. In South Carolina, Morris and Haskin (1990) found that primary production of *Spartina* was positively correlated with annual mean sea level and rainfall over a period of five years. Thus, production and patterns of dominance of *Spartina* and *Juncus* appear to differ with inundation period (flooding duration) and inundation regime (flooding pattern), although the disproportionate attention in marsh macrophyte research given to *Spartina* makes rigorous comparisons between species difficult.

The recent history of accelerating rates of sea-level rise (Kemp et al. 2011) makes need for quantification of the feedback processes that mediate marsh accretion imperative to predicting the fate of these ecosystems. Many marsh shorelines are developed with widespread use of bulkheads or revetments, which represent a physical barrier to marsh transgression (Titus et al. 2009). Consequently, the feedback processes that may allow coastal marsh persistence in situ become even more critical to understand and quantify. Research is needed to determine whether dominant marsh macrophytes occupy marsh elevations still optimal for their production or whether rising water levels have left them in conditions that already reflect probable failure in maintaining marsh surface elevations relative to sea level.

Here, we employ a field bioassay (Morris 2007; Kirwan and Guntenspergen 2012) to examine multiple metrics of plant production of the dominant macrophyte species *Spartina* and *Juncus* at a variety of elevations representing current and potential past and future conditions of rising sea level. Our objectives in this study were twofold: (1) measure and compare the growth responses of *Spartina* and *Juncus* to manipulated inundation periods, ranging from durations shorter to longer than those experienced by these macrophytes on the marsh platform; and (2) compare growth responses in *Juncus* under two differing inundation regimes (astronomically vs. meteorologically dominated flooding patterns). We then test the results against an eco-physiologically based response curve of macrophyte production (Shelford 1931; Morris et al. 2002) at each of two study sites to determine whether increasing inundation of the contemporary marsh platform would increase or decrease in situ production. Because mechanisms of marsh accretion require macrophyte production to increase with greater inundation, we use this analysis of the present status of each marsh to infer the marsh fate under a scenario of rising sea level.

## Materials and methods

### Study sites

**Fig. 1:**
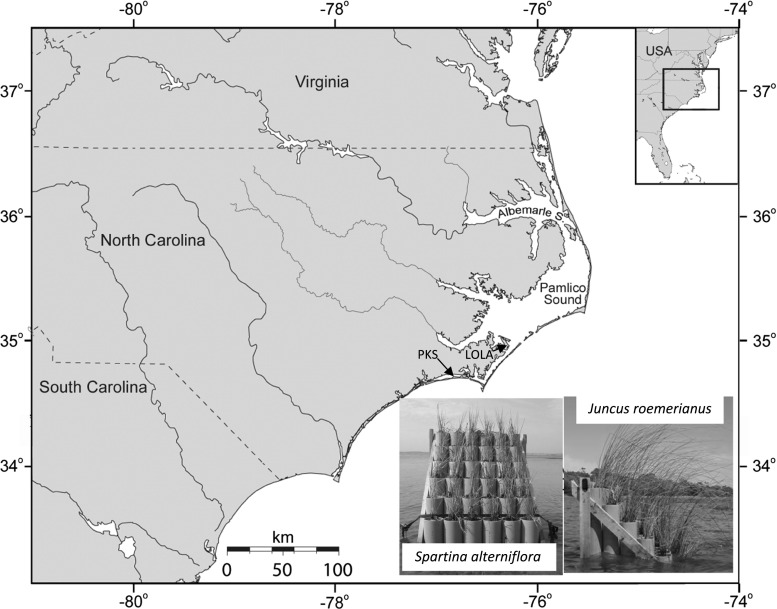
Map showing study site locations at Pine Knoll Shores (PKS) and Lola (LOLA), North Carolina and inset photographs of marsh planters containing *Spartina alterniflora* and *Juncus roemerianus*

Research was conducted at two mid-coast North Carolina (USA) sites that differ in inundation regime. Pine Knoll Shores (PKS) (33.6953°N, 76.8417°W) on Bogue Sound and Lola (LOLA) (34.9501°N, 76.2796°W) on southern Pamlico Sound are approximately 50 km apart, experience similar climatic conditions, and yet vary dramatically in hydrologic forcing due to differences in their proximity and connectivity to the Atlantic Ocean (Fig. 1). Previous measurements of water-level variation indicated an expected mean astronomical tide range of 60 cm at PKS (C. Currin of NOAA’s Center for Coastal Fisheries and Habitat Research, pers comm) and 8 cm at LOLA (CO-OPS 2004). Marsh inundation at PKS is regular, strongly forced by the semi-diurnal astronomical tide, while marsh flooding at LOLA occurs irregularly, largely in response to meteorological conditions, which can drain or flood the marsh platform for weeks at a time. *Spartina* and *Juncus* dominate the macrophyte community and form mosaics of monospecific patches across the marsh at each site, although the first 5 m of marsh edge along the estuarine shoreline is occupied almost exclusively by *Spartina* at PKS. Both the PKS and LOLA marsh sites had scarped edges facing the sound, and we often observed undercutting and slumping of ≤1-m^2^ sections of marsh edge.

### Water level recording

We established a temporary water level station at each site in accordance with the criteria of the National Oceanic and Atmospheric Administration’s Center for Operational Oceanographic Products and Services (NOAA 2007) using a dual pressure transducer system by Onset Corp (HOBO data loggers model U20-001-01). The PKS station (34.53436^o^N, 76.83176^o^W) was established at the NC Aquarium’s Bogue Sound pier in May 2006 and the LOLA station (34.95098^o^N, 76.28112^o^W) at the Lola Road U.S. Fish and Wildlife Service dock in June 2006. The North Carolina Geodetic Survey (NCGS) established a Second Order Class 2 benchmark with a known elevation relative to NAVD88 at each site near its water level station, to which we referenced benchmarks on each water-level station and at the field sites. A calibrated Topcon^®^ Model RL-50A rotating-laser system was used to determine elevations of the water-level station benchmarks, as well as of the marsh surface and elevation treatments in the experimental marsh planters, relative to the NCGS benchmark at both sites and to a temporary benchmark (nail in a tree) at PKS. We verified elevation of the temporary benchmark with a Trimble^®^ 5800 RTK GPS system unit at the PKS marsh experiment site. Knowing elevations relative to NAVD88 provides absolute elevations that can be compared broadly across geographic regions and time. We determined the relationship between NAVD88 and mean sea level (MSL) using the NOAA tidal benchmarks at Morehead City Harbor (Station ID: 8656502) for PKS and at Rodanthe, Pamlico Sound (Station ID: 8653215) for LOLA. Post-processing of water-pressure data from the always submerged transducers was completed using HOBOware^®^ to adjust for our simultaneously measured variation in time-referenced, site-specific barometric pressure. The resulting absolute water levels relative to NAVD88 and MSL were computed for an 18-month period from mid-2006 to December 2007 to document contrasting temporal patterns of inundation between sites and then to quantify inundation periods over the 2006 and 2007 experimental growing seasons for each of the six elevation levels of the marsh planters.

### Experimental marsh planters

We used planters (Morris 2007) to experimentally manipulate the elevation (and thus inundation period) of two marsh macrophytes (*Spartina* and *Juncus*) at the two study sites differing in inundation regime (Fig. 1). The lower end of each pipe rested on the estuarine bottom, and the upper end varied in elevation such that tops of each successive row of six pots extended 15 cm higher than the preceding row below, creating a range of six elevations projecting approximately 30–105 cm above the estuarine floor and −0.2–0.5 m relative to MSL, depending on row and planter (Table 1). Our use of experimental planters is designed to test the effects of differing inundation at two different sites; the experiment uses fixed site to provide an explicit, identical, and quantified inundation treatment equally to all replicate pots of plants. Replication of pots within planter is provided within each row and used to detect and measure the variability among replicate plugs of each of the two dominant marsh macrophytes. It is this variability among replicate plugs (pots) of the plants upon which statistical significance testing is based. Marsh macrophytes are also replicated using the two dominant species in the Southeast U.S. so as to assess generality of observed responses to inundation duration. Inundation regime is likewise varied by choosing one site reflective of dominance by regular astronomic tides and a second site in which meteorological forcing of inundation dominates, thereby allowing inference about inundation duration as regime changes.

**Table 1:** Elevation and percent time flooded (referenced from pot top) of all marsh planter rows

Marsh planter	Row	Elevation relative to MSL (m)	Elevation relative to NAVD88 (m)	Percent time flooded (%)	Inundation (arcsine transformed value)
PKS 2006 *Spartina*	1	−0.318	−0.200	81.0	1.120
	2	−0.180	−0.062	57.0	0.856
	3	−0.023	0.095	28.0	0.558
	4	0.099	0.217	14.0	0.383
	5	0.275	0.393	3.0	0.174
	6	0.422	0.540	0.5	0.071
PKS 2006 *Juncus*	1	−0.294	−0.176	78.0	1.083
	2	−0.150	−0.032	51.0	0.795
	3	−0.008	0.110	26.0	0.535
	4	0.144	0.262	10.0	0.322
	5	0.296	0.414	3.0	0.174
	6	0.446	0.564	0.4	0.063
LOLA 2006 *Juncus*	1	−0.316	−0.316	80.0	1.107
	2	−0.167	−0.167	53.0	0.815
	3	−0.012	−0.012	28.0	0.558
	4	0.142	0.142	24.0	0.512
	5	0.285	0.285	6.0	0.247
	6	0.452	0.452	0.7	0.084
PKS 2007 *Spartina*	1	−0.571	−0.453	99.0	1.471
	2	−0.427	−0.309	92.0	1.284
	3	−0.267	−0.149	67.0	0.959
	4	−0.135	−0.017	42.0	0.705
	5	0.014	0.132	19.0	0.451
	6	0.163	0.281	5.0	0.226

Planters were positioned identically at each field site with the entire planter in the subtidal zone just outside the marsh and facing south to avoid self-shading (Fig. 1). The elevation of the fourth row of pipes from the bottom was approximately equivalent to the mean elevation of the respective macrophyte species on the adjacent marsh platform (Table 1). At PKS, two planters were employed from March 16 to September 15 during the 2006 growing season; one planter held plugs extracted from the marsh platform of *Spartina* and the other planter of *Juncus*. Only one planter was deployed April 13 –September 21 during the 2007 season, holding three plugs of *Spartina* at each elevation. In 2007, we re-positioned the entire planter at PKS to a lower elevation, deeper than in 2006, to determine the threshold inundation period above which *Spartina* cannot survive. At LOLA, only one planter was established and only for a single growing season, March 17 to September 16, 2006, holding *Juncus* plugs in each pot with six replicates per row. The bulk of each pipe was filled with local estuarine sand, while the upper 30 cm contained the plugs within native marsh sediment. Plugs were taken from two 20-m^2^ areas of the adjacent marsh platform to maximize similarity among starting condition for above-ground standing stock, numbers of culms or leaves, environmental history, and presumptive genotype (e.g., Lessmann et al. 1997).

We compared several growth metrics of macrophytes in planter pots with those at equivalent elevations on the adjacent marsh platform to evaluate whether culturing macrophytes in pots reproduced the ecophysiological responses of unmanipulated plants in 2006. We measured total and live above-ground biomass and shoot density on the marsh platform using sampling rings cut from a planter pipe to sample six replicates haphazardly within a 1-m × 3-m plot for each possible planter-equivalent elevation. This field sampling allowed comparisons to corresponding end-of-growing-season data in the planter pots (*n* = 6 replicates). At PKS, natural expanses of *Spartina* occurred at elevations equivalent to planter rows 2–4, while *Juncus* occurred naturally at elevations matching rows 3 and 4. In 2006, row 3 *Spartina* above-ground samples (28 % inundation treatment) were lost in a laboratory accident. At LOLA, the entire marsh platform varied in elevation by only ~5 cm, allowing us to sample natural marsh control plots for only one planter row (4), but we haphazardly took six replicate samples in each of two natural *Juncus* plots of about 1-m × 3-m at the elevation matching row 4.

### Response metrics

The responses of *Spartina* and *Juncus* to mean growing-season inundation duration (via comparing elevation treatments), and *Juncus* to inundation regime (via site contrasts, holding inundation duration similar), were determined by measuring and analyzing a suite of plant growth metrics. We report ecophysiologically based responses of macrophytes over the growing season (hereafter defined as seasonal change) as net changes in: (1) total (live plus dead) above-ground biomass; (2) live (green) above-ground biomass; and (3) shoot density—culms (*Spartina*) or leaves (*Juncus*). To these responses, we added analyses of (4) end-of-season (EOS) below-ground biomass. We also compared the following: (5) EOS total above-ground biomass; (6) EOS live above-ground biomass; and (7) EOS shoot density between both planter rows and platform plots for those elevations where such comparisons are possible (see Online Resource 1). We present the information on elevations occupied by each macrophyte on natural marsh platforms at the top of the x-axis as a bold thick line, specific for each marsh planter.

To estimate the seasonal change in above-ground biomass, we first estimated initial above-ground biomass in each pot by applying species-specific length-mass regressions and then subtracted the resulting mass estimate from the observed EOS above-ground biomass. The EOS above-ground biomass was sorted into green (live) and brown (dead) fractions and treated separately to produce EOS biomass for each. Seasonal change in culm or leaf density (also an above-ground metric) was computed by direct counts in each pot at the start and end of the experimental season. Individual, sorted vegetation samples were dried to constant weight at 85 °C.

The EOS below-ground biomass was measured in each pot at the end of each experiment. The below-ground biomass from each pot was separated from sediments in a 1-mm-mesh sieve using a low-pressure wash. Individual below-ground vegetation samples were dried to constant weight at 85 °C. Four sub-samples of each sample were then ashed at 500 °C for 6 h to quantify percent organic matter content. Finally, we multiplied the average organic matter percentage from the sub-samples by the below-ground dry mass to provide EOS, ash-free below-ground biomass. The raw values of EOS total above- and below-ground biomass for all planter rows and platform plots can be found in Online Resource 2.

### Statistical analyses

SYSTAT^®^ (version 13.00.05), JMP^®^ (version 8.0) and SAS^®^ software (version 9.1) were used for statistical analyses. All data sets used to test macrophyte response variables passed O’Brien’s test of homogeneity of variance (minimum *P* ≥ 0.126) when analyzed by individual planter, each with a unique species, site, and year. Inundation period (the mean proportion of time that water level is > row elevation during a given growing season), our primary independent variable, was normalized by arcsine transformation (Sokal and Rohlf 1981). ANOVA was employed to assess the statistical significance (*α* = 0.05) of the effect of inundation on: (1) seasonal change in total (live and dead) above-ground biomass; (2) seasonal change in live above-ground biomass; (3) seasonal change in total above-ground density of shoots (culms for *Spartina* or leaves for *Juncus*); and (4) EOS below-ground biomass. Tukey–Kramer HSD was used for post hoc pairwise comparisons of means. In addition, we used the Fisher’s method of combining independent probabilities (Sokal and Rohlf 1981) as a meta-analytic technique to combine findings between years for *Spartina* and sites for *Juncus*. To address concerns that differences in initial conditions could confound results of the regression analyses of EOS below-ground biomass, we also analyzed these data with an ANCOVA, using initial total above-ground biomass as a covariate. GLM two-way ANOVAs were employed to examine the effects of inundation period, inundation regime (site), and their interaction on the growth response metrics of *Juncus*.

## Results

### Water levels

**Fig. 2:**
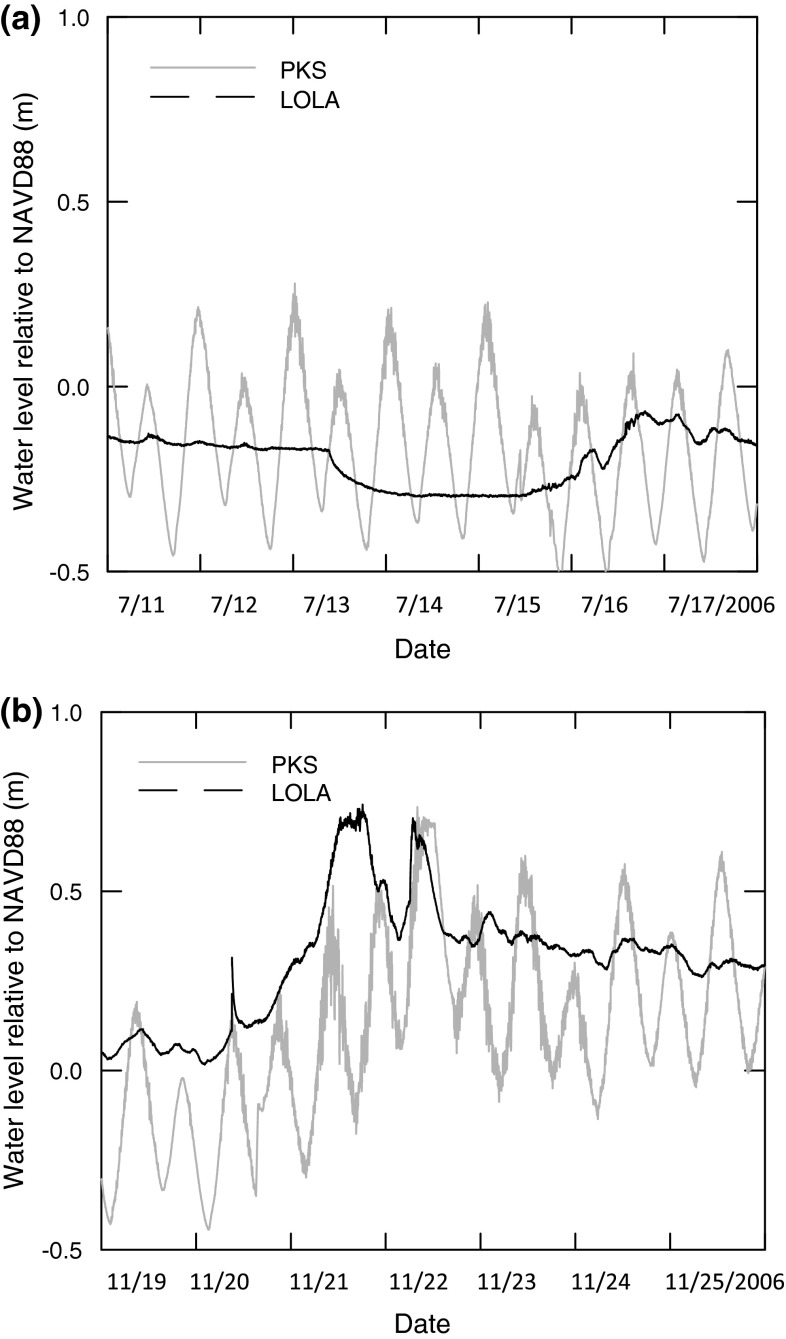
Representative 7-day water levels from the astronomically dominated site (PKS) and the meteorologically dominated site (LOLA) during summer season (**a**) and winter season (**b**) illustrating differences in inundation regimes

The 18-month water level records revealed distinctly different inundation patterns between the two sites. The primary harmonic constituents of the astronomic tide (M_2_, K_1_, O_1_, and solar annual) explained 59 % of the variance in observed water levels at PKS, but only 23 % at LOLA. Meteorological forcing presumably accounted for most of the remaining variance in water levels (41 % at PKS and 77 % at LOLA). This difference in the meteorological forcing of marsh inundation is evident in comparing how two continuous 7-day plots of water level varied between sites, one a representative period of southwest winds, typical of summer (Fig. 2a), and the other a period of northerly winds, typical of winter (Fig. 2b). The regular semidiurnal astronomical tidal cycle was evident at PKS during both 7-day periods. In contrast, LOLA, located at the southern terminus of the Pamlico Sound, exhibited long-period flooding driven by meteorological forcing from the north and lacked a strong semidiurnal signal in the water-level records. Mean estuarine surface salinity measured by refractometer at least monthly from June 2006 to September 2007 revealed a mean of 34 (±1.8 SD; *n* = 18) at PKS and 29 (±4.3 SD; *n* = 24) at LOLA.

### Vegetation in marsh planters

**Fig. 3:**
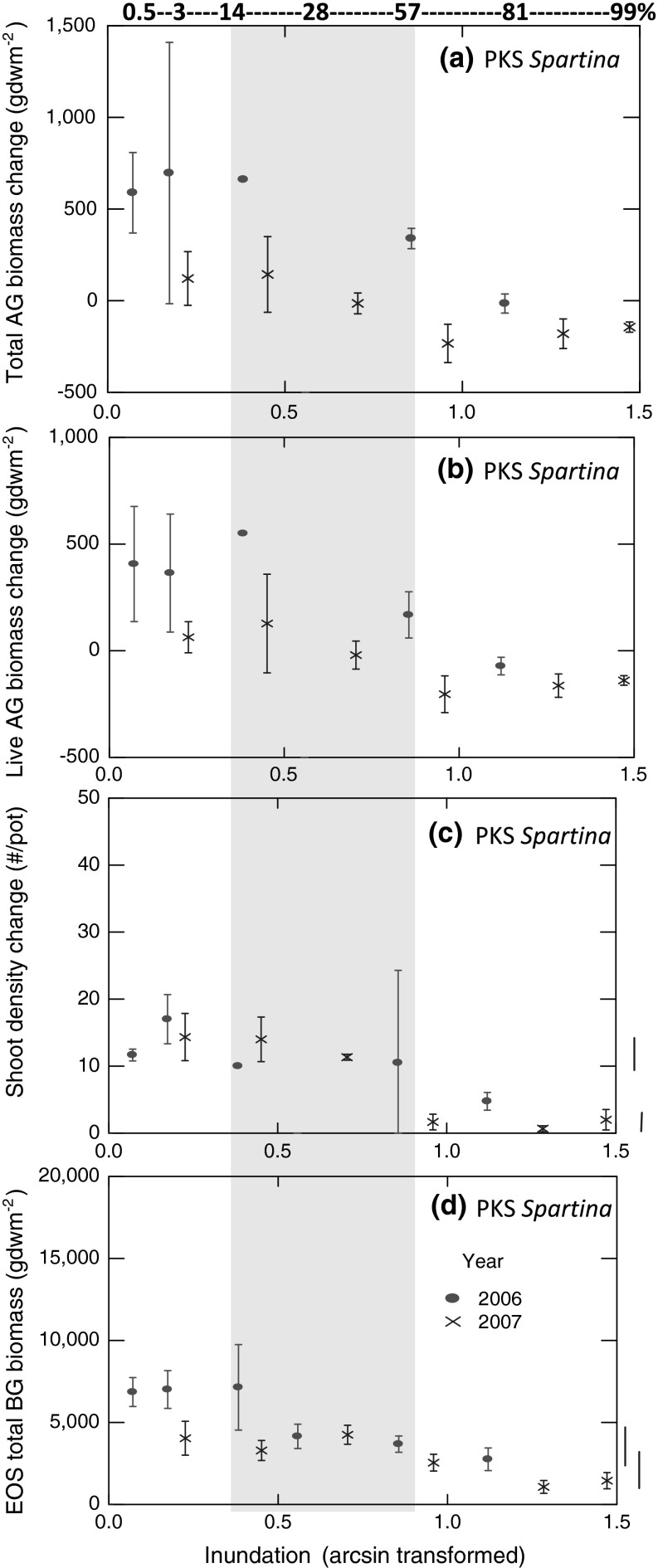
The seasonal change in **a** total (live and dead) above-ground (AG) biomass, **b** live above-ground biomass, **c** total shoot (culm) density, and **d** end-of-season (EOS) below-ground (BG) biomass of *Spartina* *alterniflora* at PKS in 2006 and 2007. Mean percent inundation period for growing season noted along top x-axis with the *shaded region* depicting the inundation experienced by *S. alterniflora* on the adjacent marsh platform for both years. *Solid lines* to *right of graphs* connect treatments that did not differ significantly in 2007, yet differed from another group using Tukey–Kramer means comparisons. No treatment groups differed significantly using Tukey–Kramer means comparisons in 2006

*Spartina alterniflora* Increasing duration of inundation produced similar patterns in the response to seasonal change in total (live plus dead) and live above-ground biomass of *Spartina* (Table 2), although the only statistically significant response detected in the ANOVAs was in total above-ground biomass in 2007 (Table 2). The green fraction of *Spartina* typically comprised about 90 % of the culm, so the similarity in responses is understandable. By combining results of both years to produce a joint significance level using Fisher’s method of combining independent probabilities, one for each year, both above-ground production metrics exhibit statistically significant responses to inundation (Table 2). While above-ground production differed little among the three treatments receiving ≤19 % inundation, greater levels of inundation resulted in progressively less growth (Fig. 3a, b). Seasonal changes in total and live above-ground biomass were less in 2007 than in 2006 for similar levels of inundation.

**Table 2:** ANOVA results for the production across 6 inundation treatments for *Spartina alterniflora* at Pine Knoll Shores (PKS) in 2006 and 2007 and *Juncus roemerianus* at PKS and LOLA in 2006 [*F*_degrees of freedom associated with the treatment, degrees of freedom associated with the error_]

*Spartina alterniflora*			*Juncus roemerianus*		
**PKS 2006**			**PKS 2006**		
Total above-ground biomass	*F*_4,7_ = 3.6968	*P* = 0.0635	Total above-ground biomass	*F*_5,30_ = 10.306	***P*** **<** **0.0001**
Live above-ground biomass	*F*_4,7_ = 3.3171	*P* = 0.0797	Live above-ground biomass	*F*_5,30_ = 4.5306	***P*** **=** **0.0034**
Shoot density	*F*_4,7_ = 2.7535	*P* = 0.1149	Shoot density	*F*_5,30_ = 4.5073	***P*** **=** **0.0035**
Below-ground biomass	*F*_5,29_ = 2.7124	***P*** **=** **0.0396**	Below-ground biomass	*F*_5,30_ = 2.7716	***P*** **=** **0.0357**
**PKS 2007**			**LOLA 2006**		
Total above-ground biomass	*F*_5,12_ = 3.1132	***P*** **=** **0.0497**	Total above-ground biomass	*F*_5,30_ = 4.1634	***P*** **=** **0.0054**
Live above-ground biomass	*F*_5,12_ = 2.5317	*P* = 0.0870	Live above-ground biomass	*F*_5,30_ = 2.9269	***P*** **=** **0.0287**
Shoot density	*F*_5,12_ = 16.310	***P*** **<** **0.0001**	Shoot density	*F*_5,30_ = 6.5352	***P*** **=** **0.0003**
Below-ground biomass	*F*_5,12_ = 7.5275	***P*** **=** **0.0021**	Below-ground biomass	*F*_5,30_ = 3.8627	***P*** **=** **0.0080**
**Fisher’s combined probability test 2 years**			**Fisher’s combined probability test 2 sites**		
Total above-ground biomass	χ_(1)_^2^ = 11.517	***P*** **=** **0.0001**	Total above-ground biomass	χ_(1)_^2^ = 28.863	***P*** **<** **0.0001**
Live above-ground biomass	χ_(1)_^2^ = 9.9427	***P*** **=** **0.0036**	Live above-ground biomass	χ_(1)_^2^ = 18.469	***P*** **=** **0.0002**
Shoot density	χ_(1)_^2^ = 22.748	***P*** **<** **0.0001**	Shoot density	χ_(1)_^2^ = 27.533	***P*** **<** **0.0001**
Below-ground biomass	χ_(1)_^2^ = 18.789	***P*** **=** **0.0002**	Below-ground biomass	χ_(1)_^2^ = 16.322	***P*** **=** **0.0002**

Bold *P* values are statistically significant (α = 0.05)

Seasonal increases in density of culms (shoots) revealed similar patterns in response to changing inundation across the two years (Fig. 3c), but only the 2007 tests revealed a statistically detectable response in ANOVAs (Table 2). Fisher’s method again demonstrated statistical significance combining the p-values of both years. Tukey–Kramer pairwise contrasts for 2007 between all possible pairs of inundation treatments revealed that responses of each of the three least-inundated treatments (5–42 %) displayed significantly (Tukey–Kramer *P* ≤ 0.01) greater seasonal increases in numbers of shoots than each of the three most-inundated treatments (67–99 %) (Fig. 3c). Although the seasonal responses in total and live above-ground biomass to increasing inundation in our experimental marsh planters became negative for the two highest inundation levels, all seasonal changes in culm density remained positive, showing net increases. Besides, demonstrating large declines over the greatest levels of inundation, each of the seasonal change metrics revealed a pattern in each year suggesting a possible, but statistically insignificant increase in production from the shortest inundation treatment to one or both of the next higher levels of inundation (Fig. 3a, b, c).

EOS below-ground biomass of *Spartina* responded significantly to varying levels of inundation in each year’s ANOVA and across both years using Fisher’s method (Table 2). Like the three above-ground metrics, EOS below-ground biomass revealed declines at the greatest levels of inundation (Fig. 3d). Only in 2007 did the use of Tukey–Kramer pairwise contrasts detect statistical significance—in this case between each of the three least-inundated treatments (5–42 %) and each of the two most inundated (92–99 %) (Fig. 3d). ANCOVAs indicated that the EOS below-ground biomass of *Spartina* did not covary in either year with the initially estimated above-ground biomass in each planter pot (*F*_1,28_ = 0.252, *P* = 0.62 in 2006; *F*_1,11_ = 0.001, *P* = 0.99 in 2007).

To summarize production responses of *Spartina,* in 2006, all three above-ground response metrics failed to show statistically significant responses to inundation in ANOVAs, but the below-ground biomass response was significant; yet all production metrics except live above-ground biomass did differ significantly in 2007. As a meta-analysis over both years, Fisher’s combined probability tests showed that all four metrics of *Spartina* production responded significantly to inundation (Table 2).

**Fig. 4:**
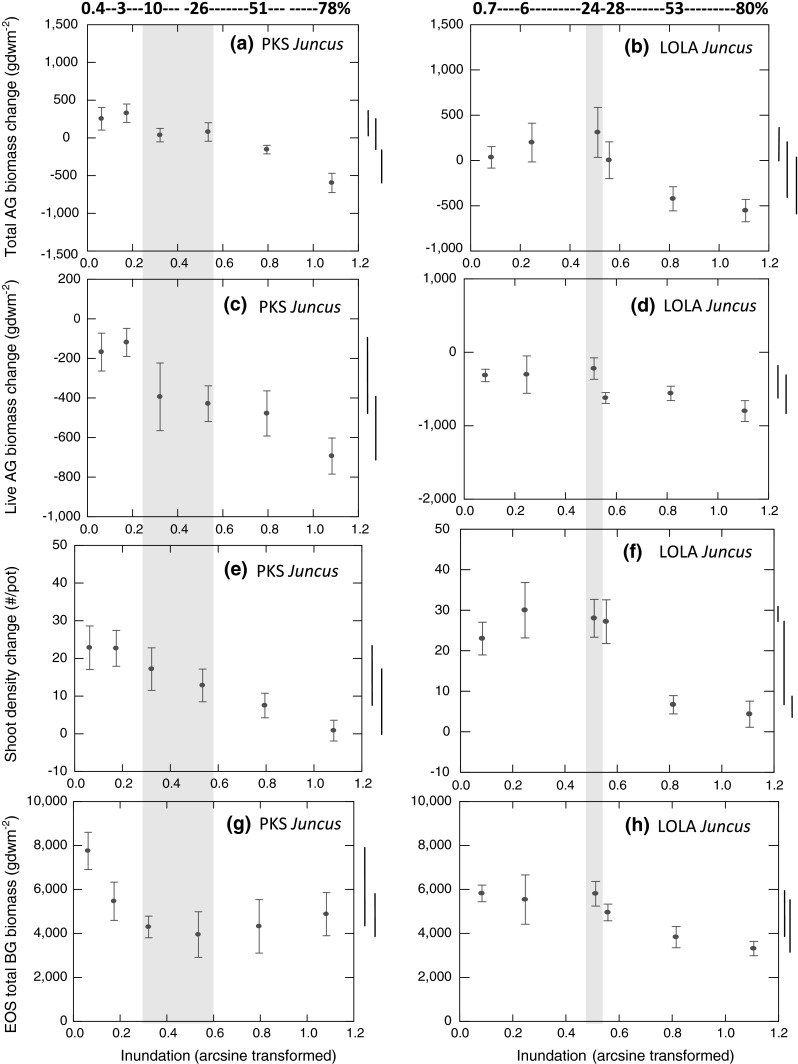
The seasonal change in **a**, **b** total (live and dead) and **c**, **d** live above-ground (AG) biomass, **e**, **f** shoot (leaf) density, **g**, **h** and end-of-season (EOS) below-ground (BG) biomass of *Juncus* *roemerianus* at PKS (*left panels*) and at LOLA (*right panels*) in 2006. Mean percent inundation periods for growing seasons noted along top x-axes with the *shaded region* depicting the inundation experienced by *J. roemerianus* on the adjacent marsh platform at each site in 2006. Treatment levels not connected by one of the *bars* to *right of graph* differ significantly using Tukey–Kramer means comparisons

*Juncus roemerianus* Duration of inundation significantly affected the seasonal change in total above-ground biomass of at both PKS (Fig. 4a) and LOLA (Fig. 4b), as detected by the ANOVAs for each site and the Fisher’s method combining probabilities from the two sites (Table 2). The pattern of response in seasonal change of total above-ground biomass resembled that of *Spartina* in suggesting an increase from the least inundated to one or more of the next two more inundated treatments, followed by a steep decline beyond an inundation of 24–26 %: this decline continued monotonically through the treatment with the longest inundation (Fig. 4a, b). Mean above-ground biomass increased over the growing season for all treatments inundated ≤26 % at PKS and ≤28 % at LOLA but displayed negative net growth for the two greatest inundations (51–80 %) at each site. Overall, this production metric for 2006 appeared lower for *Juncus* than for *Spartina*. Seasonal change in live above-ground biomass responded with a pattern similar to that of total above-ground biomass, with ANOVA detecting significant differences with changing inundation (Table 2) at PKS (Fig. 4c) and at LOLA (Fig. 4d); however, net seasonal changes in the live metric were all negative.

Seasonal change in *Juncus* leaf (shoot) density responded significantly to varying inundation in ANOVAs (Tables 2) at PKS (Fig. 4e) and at LOLA (Fig. 4f). The pattern of change in leaf density differed between the sites. At PKS, experimental manipulation of inundation produced a monotonic decrease with increased inundation across all treatments (Fig. 4e), whereas at LOLA, seasonal growth in leaf density exhibited an increase from the least inundated to the next three more inundated treatments, followed by a sharp descent to the two most-inundated treatments (Fig. 4f). Tukey–Kramer pairwise contrasts detected significant differences between some less inundated and the most-inundated treatments at each site. *Juncus* leaf density increased over the growing season at every inundation level at each site, indicating that this macrophyte, like *Spartina*, was a net producer of new shoots during the growing season despite consistent seasonal losses in total above-ground biomass at inundation treatments >26 % at PKS and >28 % at LOLA.

ANOVAs revealed that EOS below-ground biomass of *Juncus* differed significantly among inundation treatments (Table 2) at both sites. The sites did not display the same pattern of responses. At PKS, the only significant difference detected in Tukey–Kramer pairwise contrasts was the decline from the least (0.4 %) to all other greater (3–78 %) inundation treatments (Fig. 4g), whereas at LOLA, the only detectable differences were significantly greater EOS biomass at the least (0.7 %) and third least (24 %) inundated treatments than at the most (80 %) inundated treatment (Fig. 4h). ANCOVA indicated that the EOS below-ground biomass of *Juncus* did not covary with the estimated initial above-ground biomass at PKS (*F*_1,29_ = 2.694, *P* = 0.11) or at LOLA (*F*_1,29_ = 2.11, *P* = 0.16) and that the effect of inundation remained significant PKS (*F*_5,29_ = 3.0, *P* = 0.03) and at LOLA (*F*_5,29_ = 3.79, *P* = 0.009).

To summarize production responses of *Juncus,* all three above-ground metrics and the one below-ground biomass metric demonstrated a statistically significant response to inundation in ANOVAs at both sites. Accordingly, Fisher’s combined probability tests also revealed that *Juncus* production responded significantly to inundation for each of the four response metrics (Table 2).

### *Juncus* response to inundation regime

Little difference in respective *Juncus* responses was observed between the two sites, which differ by inundation regime—astronomically dominated at PKS and meteorologically dominated at LOLA. Like-numbered rows of *Juncus* grown in planters at PKS and LOLA generally experienced similar inundation periods (Table 1), allowing pairwise comparisons between sites (inundation regimes) while holding average inundation treatment constant. GLM two-way ANOVAs showed that seasonal change in total and in live above-ground *Juncus* biomass differed by duration of inundation (each *P* < 0.0001; *F*_1,68_ = 40.317 total, *F*_1,68_ = 26.913 live), not by site (*F*_1,68_ = 0.142, *P* = 0.71 total; *F*_1,68_ = 0.334, *P* = 0.56 live), with no significant interactions for either the total (*F*_1,68_ = 0.105, *P* = 0.75) (Fig. 4a vs. b) or live (*F*_1,68_ = 0.010, *P* = 0.92) (Fig. 4c vs. d) metric. Analogous testing showed that the seasonal change in shoot density differed by inundation duration (*F*_1,68_ = 48.717, *P* < 0.0001) and site (*F*_1,68_ = 8.409, *P* = 0.005), without interaction (*F*_1,68_ = 1.333, *P* = 0.25), and revealed generally greater increases in leaf densities in planters at LOLA (Fig. 4e vs. f). GLM two-way ANOVA showed that EOS below-ground biomass differed by inundation duration (*F*_1,68_ = 17.126, *P* = 0.0004), but not by site (*F*_1,68_ = 0.733, *P* = 0.78), with no interaction (*F*_1,68_ = 0.106, *P* = 0.63) (Fig. 4g vs. h).

## Discussion and conclusions

Employing experimental planters, we demonstrated remarkably similar patterns of how production metrics respond to varying inundation for two dominant macrophytes, *Spartina* and *Juncus*, and for *Juncus* across two sites differing in inundation regime, permitting important conclusions about resilience of the coastal marsh habitat to sea-level rise. Metrics of change over the growing season in total and live above-ground biomass and in shoot density all exhibited sustained and statistically significant declines with increasing inundation beyond an apparent optimal range of inundation levels (Figs. 3a–c, 4a–f; Table 2). Across the shortest two or three experimental inundation periods, both macrophytes exhibited indications of increasing production with increasing inundation during the growing season. Although none of these suggested increases was statistically significant, 10 of 12 above-ground metrics of seasonal growth for macrophyte species, years, and sites revealed the early rise (Figs. 3a–c, 4a–f). Thus, our range of experimental inundations appears to include sub-optimal conditions for both species at the low end and the high end of inundation duration. Such a parabolic response was proposed by Morris et al. (2002) (see Fig. 2) and is based upon the fundamental concept in ecophysiology known as the law of tolerance (Shelford 1931).

By incorporating a sufficient range of marsh surface elevations in our planters, we tested elevations now occupied by macrophytes on the existing natural marsh platform, plus elevations both higher and lower. The lower elevations anticipate environmental conditions associated with future sea-level stands unless the marsh surface accretes vertically at a rate at least equal to that of relative sea-level rise. The Morris et al. (2002) model reasonably assumes that the relationship between inundation and production of a marsh macrophyte follows a parabolic curve in which production decreases on either side of peak production over some optimal range of inundations. If a given marsh is positioned on the ascending (left-hand) side of the curve, where inundation is below optimal levels, then rising sea levels will increase inundation, resulting in enhanced above-ground and below-ground production. Increasing below-ground production causes accretion directly by subsurface addition of organic material (bioaccumulation), while higher above-ground macrophyte biomass leads to greater baffling of tidal water flows, thereby inducing greater sedimentation. Hence, the enhancement of macrophyte production as water levels rise within this region of the parabola represents a compensatory feedback process that could allow the marsh surface accretion to equilibrate with rising sea level (Morris et al. 2002). An additional regulatory feedback process has been demonstrated by Fragoso and Spencer (2008), who found that *S. anglica* production was positively related to burial of the basal meristem by sediments. Although these feedback processes elevate the sediment surface as sea level rises, whether the marsh surface is elevated rapidly enough to match the growing rates sea-level rise and avoid ultimate physiological drowning of the macrophytes is unclear. Furthermore, the rate of sedimentation onto the marsh surface is also affected by other factors, such as sediment concentrations in the water column, duration and frequency of tidal flooding, and volume of water in the tidal prism. In contrast to conditions that characterize locations on the left-hand, ascending portion of the parabola relating macrophyte production to inundation, for a marsh positioned on the right-hand, descending portion of the curve, increased inundation from rising sea levels decreases marsh plant production. This reduces net sedimentation and bioaccumulation and leads to drowning of the marsh macrophytes.

**Fig. 5:**
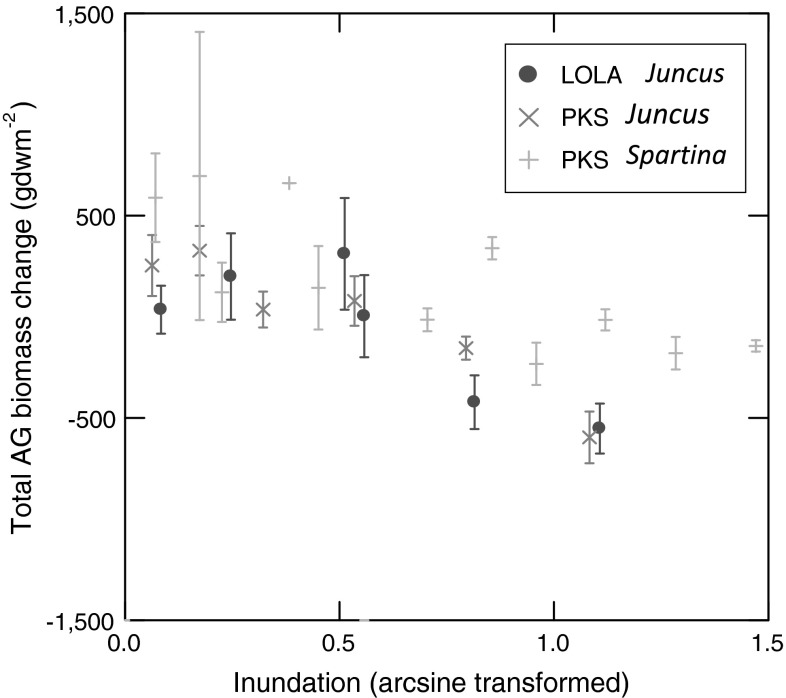
A *combined* *plot* showing the seasonal change in total above-ground biomass by inundation period for all marsh planters: *Spartina alterniflora* and *Juncus roemerianus* at Pine Knoll Shores, and *J. roemerianus* at Lola, NC

Our planter experiments testing the consequences of varying inundation levels on two marsh macrophytes in two central North Carolina coastal marshes provide data that confirm the validity of the Morris et al. (2002) model (Fig. 5). Assessing where elevations actually occupied by marsh macrophytes on the adjacent natural marshes fall on the parabolic curve traced out by our empirical growth data provides insight into the general ability of marshes in the central coastal region of North Carolina to maintain themselves in situ as sea level rises.

Despite the apparent existence of ecophysiological and sedimentary feedback processes that Morris et al. (2002) predicted, the elevations and thus inundation levels at which *Spartina* and *Juncus* actually occur on the adjacent natural marsh platforms at our study sites do not fall on the ascending (left-hand) side of the production curve. In most cases, the existing inundation levels at which both marsh macrophytes are found are located on the descending portion of the curve beyond the peak production (Figs. 3 and 4). This indicates that the marsh is unstable because, as water levels rise further, the macrophytes will respond with reduced productivity and therefore reduced capacity even to retain existing marsh surface elevations. We infer that both below-ground production and induction of sedimentation through baffling by above-ground macrophyte biomass are declining as sea level rises for both our study marshes.

Our capacity to generalize from these conclusions about the precarious status marshes in central North Carolina is limited by insufficient knowledge of factors determining sediment delivery to the marshes. Where sediment concentrations in the water column and accordingly rates of sediment delivery to the marsh are higher, marsh macrophytes can achieve more sedimentation and thereby facilitate higher rates of accretion (Kirwan et al. 2010). Without a sufficient sediment supply, even marshes with high above-ground standing stock biomass may fail to trigger the magnitude necessary in the positive feedback response to enable the marsh surface to equilibrate to rising sea levels via sedimentation. Sediment concentrations in the estuarine waters of Pamlico Sound region of North Carolina are generally low (Wells and Kim 1989; Lunetta et al. 2009) compared to those of the Chesapeake Bay (Hobbs et al. 1992) and North Inlet, South Carolina (Vogel et al. 1996), for example. Below-ground bioaccumulation may be the dominant mechanism whereby marshes must accrete in low-sediment waters (Turner et al. 2000, Nyman et al. 2006). However, we found that, like above-ground production, below-ground production generally also decreased with increasing inundation beyond a given threshold (Figs. 3d, 4g, h). Our results conform to findings of Blum (1993) and Blum and Christian (2004), who demonstrated that macrophyte below-ground biomass was greatest at higher elevations. Also, Blum (1993) found that bioaccumulation contributions account for a greater proportion of vertical accretion in high-marsh zones. In a study that used multi-level marsh planters to examine *Schoenoplectus americanus* and *Spartina patens* production response to elevation (inundation), Kirwan and Guntenspergen (2012) demonstrated that the optimum elevations for above- and below-ground production might differ for a given species. Our results show that above- and below-ground production of each macrophyte species that we assessed apparently share optimum elevations. Whether marsh accretion is dominated by sedimentation or bioaccumulation, both processes depend upon macrophyte production that increases with increasing inundation under a scenario of rising sea level.

The frequency with which the volume of sediment-laden waters moves in over the marsh surface also plays an important role in sedimentation. For astronomically dominated tidal inundation regimes, the diurnal or semi-diurnal tidal prism renews the sediment load of overlying waters on the marsh either once or twice daily. In contrast, meteorologically dominated marsh platforms may remain continuously flooded or dry for days or even weeks without renewal of sediment loads from sediment-laden estuarine water sources. Frequency of marsh flooding, and importation of new sediments, thus affects the magnitude of surface sedimentation, such that marshes experiencing meteorologically dominated inundation are likely to receive less net sedimentation than those with identical tidal prisms that are replaced more frequently. Nevertheless, intense storms can be responsible for a large fraction of the annual sedimentation on coastal marshes (Leonard et al. 1995; Reed 2002). Such events may provide compensatory accretion in a manner not evaluated here.

One study objective was to assess the effect of varying inundation regime on *Juncus* growth. While others have examined how inundation duration influences *Juncus* production (Christian et al. 1990; Tolley and Christian 1999; Pennings et al. 2005), to our knowledge, this contrast of inundation regimes on *Juncus* growth is unique. Because our two study sites were geographically close and thus experienced similar environmental conditions, differing somewhat in salinity but exposed to similar climatic conditions, the major factor that may induce differences in marsh macrophyte production is presumably the flooding regime (Minello et al. 2012). In our planters, we successfully produced almost identical durations of inundation between PKS and LOLA for each corresponding planter row, allowing us to test whether substantial differences in the temporal pattern of flooding induced different macrophyte production responses (Table 1). The patterns of change in the suite of production metrics across varying levels of inundation and even the quantitative levels of the metrics at corresponding inundations were surprisingly similar despite the radical differences in inundation regime (Fig. 2). Only the seasonal change in shoot density of *Juncus* differed detectably with site (inundation regime), being lower at PKS with its regular astronomical flooding regime than at LOLA with its meteorological flooding pattern.

Bertness (1991), Bertness and Pennings (2000), and Pennings et al. (2005) present observations and experimental results characterizing *Juncus* (*roemerianus* and *gerardii*) as a macrophyte genus that is restricted to the high marsh and physiologically prevented by inundation period and salinity from extending lower on the shore where *Spartina* dominates. Our experimental assessment, however, of how *Juncus* and *Spartina* respond to varying inundation duration at PKS revealed essentially indistinguishable responses, although our resolution of potential differences between species is coarse because we spread our six inundation treatments over a wide range at 15 cm intervals. In addition, the accidental loss of all above-ground samples of *Spartina* from the 2006 sampling of row 3 in the PKS planter, which experienced a 28 % inundation, prevents us from knowing if *Spartina* and *Juncus* initiated their performance declines over an identical range of between 3 and 28 % inundation (we must now report the range as 3–19 % for *Spartina*, lacking data for the 28 % treatment). Nevertheless, the similarity between the two species of marsh macrophyte in their production responses to varying duration of inundation is striking (Fig. 5). This similarity suggests that some other factor besides inundation may prevent *Juncus* from extending to lower levels on marsh platforms.

*Juncus* is known to grow abundantly down to the estuarine edge of marshes on relatively quiescent shorelines of Albemarle, Currituck, and Pamlico Sounds in North Carolina (Wilson 1962; Brinson 1991) and along the Gulf of Mexico (Stout 1984). Field experiments by Bertness and Ellison (1987), Bertness (1991), and Pennings et al. (2005) showed that interspecific competition plays a significant role in creating vertical zonation patterns between different species of marsh macrophyte, including species of *Spartina* and *Juncus*. These previous studies revealed substantial overlaps in physiological tolerances of many marsh macrophytes, with biological interactions explaining important aspects of spatial segregation. Others have found that the net annual primary productivity, leaf longevity, and decomposition rates of *Juncus* differ little over a range of hydroperiods (Christian et al. 1990; Tolley and Christian 1999). However, disturbance, such as that from wrack deposition (Brinson and Christian 1999; Tolley and Christian 1999) or fire (Schmalzer et al. 1991), appears to drive declines in or absence of *Juncus* biomass where tidal inundation was frequent.

Because the use of a field intervention such as the marsh planter may create growing conditions that differ from the natural marsh platform, we tested for evidence of potential planter artifacts. Possible contrasts were limited by our intentional inclusion of inundation levels outside the range that occurred on the marsh platform: this reduced both the number and range of inundation treatments available for comparison. Tests for potential artifacts of culturing marsh macrophytes within planter pots revealed one out of five contrasts of EOS live above-ground biomass and one of five contrasts for shoot density that differed significantly (higher values of above-ground biomass and lower values of shoot density on the natural marsh platform: see Online Resource 1 Fig. S1). We also tested for evidence of an interaction between this putative artifact effect and our inundation treatment effect (see Peterson and Black 1994). We detected no significant interaction, but each graph of the magnitude of the putative artifact exhibited a decline with inundation (see Online Resource 1 Fig. S2). Nevertheless, even if this non-significant pattern was real and we were to adjust the magnitudes of treatment effects by subtracting away the putative artifact, a substantial treatment effect would remain (see Online Resource 1).

Our assessment of the ecophysiological status of the dominant macrophytes in two North Carolina marshes differing in inundation regime revealed evidence strongly suggesting that both macrophytes are drowning under present inundation levels and that the physiological stress of inundation will only increase as sea level continues to rise. Our ability to generalize beyond these two marshes in central North Carolina depends upon how representative their current rate of relative sea-level rise is of other geographic areas. Based upon NOAA tide gauge stations with records exceeding 50 years, the mid-Atlantic region from New York to North Carolina exhibited comparatively high rates of relative sea-level rise at 1.75–4.42 mm year^−1^ (Zervas 2001). The Gulf Coast from Louisiana to Texas showed even higher rates of relative sea-level rise at 3.38–9.85 mm year^−1^, whereas the south Atlantic rates ranged from 2.04 to 3.28 mm year^−1^ (Zervas 2001). Consequently, if responses of these macrophyte species are similar across regions, marsh macrophytes from Louisiana through Texas may be drowning at even faster rates than those of central North Carolina, assuming no large effects of differing sediment delivery rates. We acknowledge that both duration of tidal inundation and suspended sediment concentrations in an estuary are also important factors that influence changes in marsh surface elevation with sea-level flux (Kirwan et al. 2010; D’Alpaos et al. 2011). Another important consideration when assessing marsh sustainability is that marshes, even those with similar vegetational community composition, occur naturally over a range of elevations (McKee and Patrick 1988) and that elevation relative to sea level influences marsh vulnerability to sea-level rise (Cahoon and Guntenspergen 2010). Geographically, those regions experiencing the greatest risk of loss of marsh habitat area share a high rate of relative sea-level rise and occupation of a broad area of low-sloped topography. Specifically, (Titus and Richman 2001) predict highest rates of marsh loss to occur in the Mississippi Delta, South Florida, and Northeast North Carolina.

If in situ persistence of coastal marshes is unlikely in central North Carolina and other geographic areas characterized by high rates of relative sea-level rise, transgression landward remains the mechanism that could allow these marshes to continue to provide their valuable ecosystem services indefinitely into the future. Transgression, which sustained coastal marshes during historical periods of sea-level rise (Redfield 1965; Orson et al. 1987; Reed 2002), is itself challenged by widespread installation of bulkheads and rock revetments to prevent erosion and protect shoreline development (Titus and Craghan 2009). In the presence of such engineered barriers, coastal marsh is squeezed between a fixed barrier and the rising estuarine waters, leading to habitat loss, while the bulkhead wall prevents transgression (Peterson et al. 2008). Novel solutions to this policy challenge are urgently needed, perhaps involving the implementation of rolling easements that require stepwise retreat from the estuarine edge (Titus 1998) or the de-embankment of the hardened estuarine shoreline (Wolters et al. 2005).

## Electronic supplementary material

Below is the link to the electronic supplementary material.

Supplementary material 1 (PDF 318 kb)

Supplementary material 2 (PDF 98 kb)
