# Supplementary material for: Marsh macrophyte responses to inundation anticipate impacts of sea-level rise and indicate ongoing drowning of North Carolina marshes
Source: Mar Biol. 2012 Oct 17;160(1):181–94. doi: 10.1007/s00227-012-2076-5 (PMC3873026; doi:10.1007/s00227-012-2076-5)
Supplement: Supplementary file 1 — Supplementary material 1 (PDF 318 kb) [file 227_2012_2076_MOESM1_ESM.pdf]

Marsh macrophyte responses to inundation anticipate impacts of sea-level rise and indicate ongoing drowning of North Carolina marshes

Christine M. Voss[1,*] • Robert R. Christian[2] • James T. Morris[3]

[1]East Carolina University, Coastal Resources Management, Greenville, NC 27858, USA
Current address: University of North Carolina at Chapel Hill, Institute of Marine Sciences, Morehead City, NC 28557, USA [*] e-mail: christinemvoss@gmail.com; cvoss@unc.edu
[2]East Carolina University, Department of Biology, Greenville, NC 27858, USA
[3]Department of Biological Sciences and the Belle W. Baruch Institute for Marine and Coastal Sciences, University of South Carolina, Columbia, SC 29208, USA

Online Resource 1. Planter-platform comparisons: examining possible artifact effects

Methods

Although marsh planters have been used elsewhere to examine macrophyte production experimentally (see text) over an extended range of inundation levels, we found no cases in which the performance of macrophytes cultivated in planters was compared with those grown *in situ*. Although experimental planters may induce artifacts, they are a creative and necessary intervention that allows tests of how marsh macrophytes respond to inundation levels outside the range under which they live currently on the marsh platforms. Our experimental procedure involved considerable manipulation of the plants. Differences may exist between conditions on the natural marsh and the experimental pots at the same elevation. Thus, we tested for potential artifacts associated with the experiment by making comparisons between plants on the marsh platform and planter pots at equivalent elevations. Only some planter treatments possessed a corresponding marsh platform inundation, limiting the number of possible comparisons. In addition, our most rigorous tests of inundation effects employ metrics based upon change during the experiment and such seasonal change data are unavailable for plants on the marsh platform, limiting us to examining potential artifacts on end-of-season (EOS) metrics only. We employed two different types of tests for evidence of potential artifacts. First, we used two-sample t-tests (or in one case a 1-sample t-test where no replication existed from the planter because of sample losses) on EOS above-ground biomass, EOS live above-ground biomass, and EOS shoot density, on differences in treatment means as an indication of possible artifacts. Second, for each of these same three metrics of plant production, we computed the difference between treatment means of the marsh planter and corresponding marsh platform plot (as the best estimate of magnitude of an artifact,

if one existed), and regressed the set of these differences against inundation period, testing whether the estimated artifact varied significantly with inundation, our independent treatment variable (Peterson and Black 1994).

Results

Five comparisons of EOS production metrics could be made between macrophytes in the planter pots and at corresponding inundation levels on the marsh platform. We plot the five available data points (treatment means ± 1 std error) from measurements made on macrophytes in the natural marsh on graphs that simultaneously depict not only the metrics from the five corresponding planter treatments, but also the pattern of change in those metrics across all inundation treatments (Fig. S1). For EOS total above-ground biomass (Figs. S1 a-c), EOS live above-ground biomass (Figs. S1 d-f), and EOS shoot density (Figs. S1 g-i), the mean values from the marsh platform generally fell within the range of planter pot values across the inundation treatments. The apparent pattern of response to inundation period in these EOS metrics from the planter experiment broadly matched the trends in the seasonal change indices that we formally tested, with all three production metrics declining with greater inundation (Fig. S1). Results of t-tests comparing metric means between planter pots and marsh platform for each of the five matched pairs revealed no statistically significant difference in EOS total above-ground biomass. In one of the five tests, EOS live above-ground biomass revealed a significant difference, with the planter mean higher than the marsh platform mean for a *Juncus* treatment at PKS (Fig. S1 e). The EOS shoot density appeared to be generally higher in planter pots than for the corresponding inundation level in the marsh platform, but only one of the five pairs (*Juncus* at LOLA) demonstrated a statistically significant difference (Fig. S1 i). Fisher's method of combining independent probabilities (Sokal and Rohlf 1981) across all five t-test comparisons revealed significantly higher levels of each production metric in the planter pots as compared to the corresponding inundation treatment in the natural marsh (Fig. S1). This composite test combines sites and macrophyte species, although all from 2006.

Regressing putative artifact size (the difference between the planter mean and the natural marsh mean) of each of the 3 EOS metrics against inundation treatment, again using just the five possible comparisons available, failed to reveal a statistically significant slope for any metric (Fig. S2 a-c). Nevertheless, this test has low power with only five data points and each graph for EOS total above-ground biomass, live above-ground biomass, and shoot density depicts an apparent pattern of decline in the metric with increasing inundation.

Discussion

Results of tests for potential artifacts of culturing marsh macrophytes within planter pots revealed evidence that each production metric may have been higher in the planter than at the corresponding elevation on the natural marsh platform (Fig. S1). This pattern was especially strong for the analysis of EOS density of shoots, based upon combining results of all five contrasts between planter pots and corresponding plots on the marsh platform. These tests are not especially compelling because, simply to provide replication, we necessarily combined contrasts across macrophyte species and study site. We nonetheless propose two explanations for the possible enhancement of shoot density in the planter pots. First, the higher EOS shoot density in the planter pots could be a stress response induced by a more energetic environment ("wave pruning") in the planters that were positioned just offshore of the natural marsh platform, perhaps analogous to a *Distichlis spicata* density increase in response to mowing (de Szalay and Resh 1997). Second, the area of each plug of soil and macrophytes that we introduced into the planter pots was slightly smaller than the surface area available in a pot, offering more opportunity for subsequent emergence of new shoots, especially near the edge where unvegetated soil was placed.

The most serious consequence of experimental artifacts arises if the magnitude of an artifact is not constant but changes with experimental treatment: then contributions from the pattern of how the artifact changes across treatments could confound detection of an actual treatment effect (Peterson and Black 1984). To provide insight into the possibility of a planter artifact varying with the inundation treatment, we again employed the only data available, plotting the EOS differences between the marsh platform plots and planter pots for 3 production metrics - total above-ground biomass, live above-ground biomass, and shoot density. The resulting lack of statistical significance in each of these regressions may imply that the magnitude of a possible planter artifact does not vary with inundation (Fig. S2).

We also examined how each of these three EOS metrics changed in the planter pots over the range of inundations included in the plots of observed differences between pot and marsh platform metrics (Fig. S2). We repeated the linear regression analyses combining points for both species and both study sites in 2006 and use the resulting slope as a measure of the strength of the relationship of the response that would combine potential artifacts with real treatment effects. For EOS total above-ground biomass the negative slope was 100% and for EOS shoot density 50% greater in this response that combines potential artifacts with true effects of inundation than for putative

artifacts alone, implying that even after subtracting putative artifacts, a true treatment effect exists. For EOS live above-ground biomass, the slope of the joint effect of artifacts and true treatment effects was 18% less than that of potential artifacts alone. We used in our presentations of results the more rigorous seasonal change metrics in our analyses of inundation effects on these three metrics, not the EOS measures, and artifacts associated with the seasonal changes metrics may not follow those for EOS measures. In brief, using tests of limited power, we assessed artifacts related to culturing macrophytes in planter pots and these suggested higher production than occurred among macrophytes at matching elevations on the marsh platform. Regressions of whether potential artifacts varied with inundation treatment revealed declines in putative artifact with inundation, in two of the three tests, but this non-significant relationship was generally weaker (showed a less steep slope) than the reported treatment effects, implying that a residual treatment effect would remain even after removal of the putative artifacts.

References

de Szalay FA, Resh VH (1997) Responses of wetland invertebrates and plants important in waterfowl diets to burning and mowing of emergent vegetation. Wetlands 17:149-156

Peterson CH, Black R (1994) An experiment's challenge: when artifacts of intervention interact with treatments. Mar Ecol Prog Ser 111:289-297

Sokal RR, Rohlf FJ (1981) Biometry- the principle and practice of statistics in biological research, 2nd edition. W.H. Freeman and Co, New York

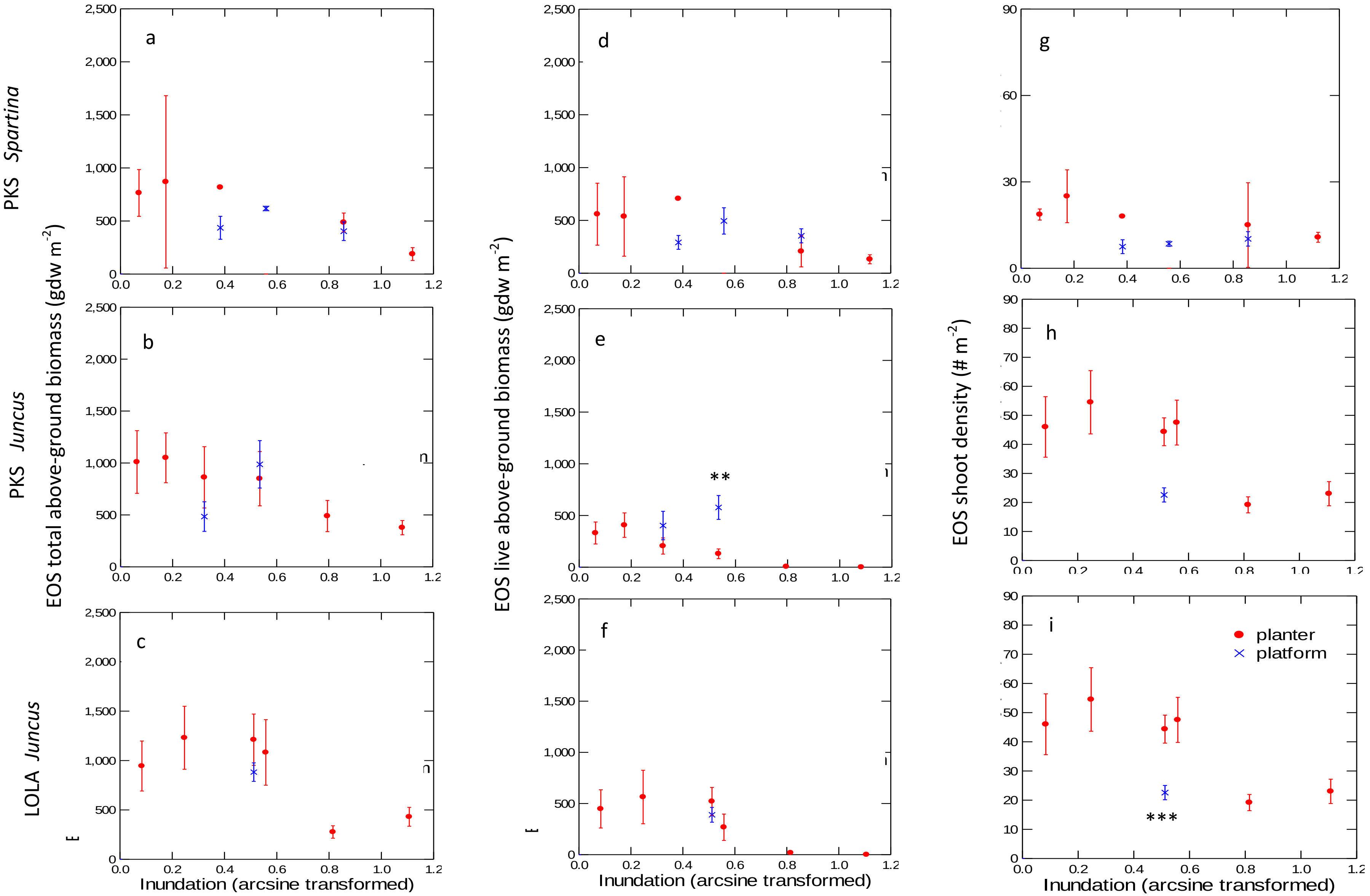

Fig. S1 End-of-season (EOS) marsh macrophyte response to inundation in planters (red dots) and on platform (blue x) comparing: total above-ground biomass of (a) *Spartina* at PKS, (b) *Juncus* at PKS and (c) *Juncus* at LOLA; live above-ground biomass of (d) *Spartina* at PKS, (e) *Juncus* at PKS and (f) *Juncus* at LOLA; and shoot density of (g) *Spartina* at PKS, (h) *Juncus* at PKS and (i) *Juncus* at LOLA ** indicates pooled variance for treatment P = 0.003 and *** indicates pooled variance for treatment P = 0.0002. Fisher's combined probability test for the 5 individual tests yielded: P = 0.04 for EOS total above-ground biomass; P = 0.02 for EOS live above-ground biomass; and P = 0.0005 for EOS shoot density

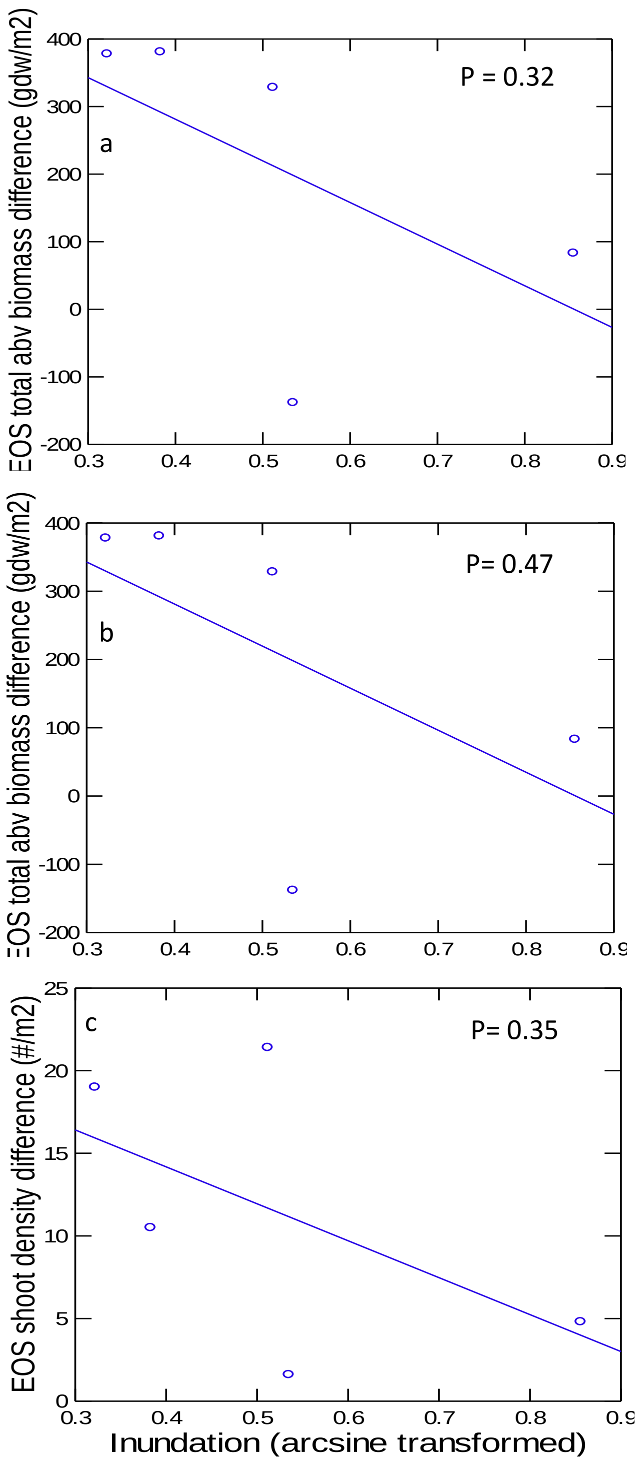

Fig. S2 Comparisons of planter and platform end-of-season metrics showing planter – platform differences in: (a) total above-ground biomass; (b) live above-ground biomass; and (c) shoot density P-values indicate non-significant slopes
