# Supplementary material for: Marsh macrophyte responses to inundation anticipate impacts of sea-level rise and indicate ongoing drowning of North Carolina marshes
Source: Mar Biol. 2012 Oct 17;160(1):181–94. doi: 10.1007/s00227-012-2076-5 (PMC3873026; doi:10.1007/s00227-012-2076-5)
Supplement: Supplementary file 2 — Supplementary material 2 (PDF 98 kb) [file 227_2012_2076_MOESM2_ESM.pdf]

Marsh macrophyte responses to inundation anticipate impacts of sea-level rise and indicate ongoing drowning of North Carolina marshes

Christine M. Voss[1,*] • Robert R. Christian[2] • James T. Morris[3]

[1]East Carolina University, Coastal Resources Management, Greenville, NC 27858, USA
Current address: University of North Carolina at Chapel Hill, Institute of Marine Sciences, Morehead City, NC 28557, USA [*] e-mail: cvoss@unc.edu
[2]East Carolina University, Department of Biology, Greenville, NC 27858, USA
[3]Department of Biological Sciences and the Belle W. Baruch Institute for Marine Biology and Coastal Research, University of South Carolina, Columbia, SC 29208, USA

Online Resource 2. Summary of above- and below- ground End-Of-Season (EOS) total biomass showing mean, standard error of the mean (SE) and number of replicates (*ŋ*) for *Spartina* and *Juncus* marsh planter rows at Pine Knoll Shores (PKS), North Carolina in 2006 and 2007, and for the *Juncus* marsh planter rows at Lola, North Carolina in 2006 (see main text for inundation treatment for each row, unique for each planter)

	row	Mesocosm total aboveground biomass mean (SE)	*ŋ*	Mesocosm total belowground biomass mean (SE)	*ŋ*	Marsh platform total aboveground biomass mean (SE)	*ŋ*
PKS 2006 *Spartina*	1	188.38 (50.6)	4	2755.38 (622.3)	6		
	2	486.5 (48.1)	2	3676.9 (445.0)	6	403.48 (76.96)	5
	3	-	0	4150.1 (648.9)	6	617.65 (11.55)	2
	4	816.8	1	7138.7 (2350)	6	435.97 (97.46)	6
	5	868.7 (441.9)	2	7011.2 (1037)	5		
	6	764.3 (166.9)	3	6853.1 (793.6)	6		
PKS 2006 *Juncus*	1	377.2 (61.7)	6	4874.6 (881.4)	6		
	2	489.0 (135.2)	6	4321.1 (1096)	6	-	
	3	848.9 (235.5)	6	3948.3 (935.2)	6	987.07 (205.65)	6
	4	861.8 (265.7)	6	4294.5 (442.7)	6	484.00 (77.55)	2
	5	1050.2 (216.2)	6	5462.8 (784.0)	6		
	6	1009.7 (271.8)	6	7753.1 (762.2)	6		
LOLA 2006 *Juncus*	1	430.3 (85.9)	6	3312.7 (295.1)	6		
	2	276.5 (56.8)	6	3831.2 (434.6)	6		
	3	1082.4 (299.0)	6	4953.9 (342.5)	6		
	4	1211.2 (233.8)	6	5807.0 (504.7)	6	993.47 (64.7)	6
	5	1230.6 (287.5)	6	5538.7 (1008.9)	6		
	6	944.4 (228.0)	6	5819.0 (341.2)	6		
PKS 2007 *Spartina*	1	6.33 (1.38)	3	1448.5 (374.0)	3		
	2	5.5 (1.59)	3	1068.5 (296.1)	3		
	3	69.0 (9.08)	2	2545.6 (389.0)	3		
	4	301.3 (9.55)	3	4247.8 (438.6)	3		
	5	351.3 (206.2)	3	3289.7 (461.4)	3		
	6	382.6 (148.6)	3	4035.4 (781.2)	3		
